# Sensitization of glycoengineered interferon-β1a-resistant cancer cells by cFLIP inhibition for enhanced anti-cancer therapy

**DOI:** 10.18632/oncotarget.14573

**Published:** 2017-01-10

**Authors:** Tae-Eun Kim, Sungyoul Hong, Kyoung Song, Sang-Ho Park, Young Kee Shin

**Affiliations:** ^1^ College of Pharmacy and Research Institute of Pharmaceutical Sciences, Seoul National University, Seoul 08826, Republic of Korea; ^2^ Abion Inc., R&D Center, Seoul 08394, Republic of Korea; ^3^ GE Healthcare Korea, R&D Center, Incheon 21988, Republic of Korea

**Keywords:** IFN-β resistance, R27T, cFLIP, sensitization, anti-cancer therapy

## Abstract

In this study, we examined the molecular mechanism underlying the resistance of cancer cells to R27T, a glycoengineered version of recombinant human interferon (IFN)-β1a, and sought to overcome R27T resistance through combination therapy. R27T has been shown to induce anti-proliferation and apoptosis in human OVCAR-3 and MCF-7 cells, but not in HeLa cells. R27T treatment increased caspase-8 activity and the consequent cleavage of caspase-8 and -3 in R27T-sensitive OVCAR-3 cells, but not in R27T-resistant HeLa cells. Conversely, R27T increased the expression of cellular FLICE-like inhibitory protein (cFLIP) in HeLa cells, but not in OVCAR-3 cells. The sensitization of HeLa cells with cFLIP small interfering RNA or 4,5,6,7-tetrabromobenzotriazole (TBB, an inhibitor of casein kinase-2) facilitated R27T-induced caspase activation, and consequently apoptosis. In OVCAR-3-xenografted mice, intraperitoneal administration of R27T showed 2.1-fold higher anti-tumor efficacy than did the control vehicle. The combined administration of R27T and TBB showed the greatest anti-tumor effect in HeLa tumor-bearing mice, reducing the relative tumor volume by 35.7% compared to that in R27T-treated mice. Taken together, our results suggest that R27T has potential as an anti-cancer drug, and combination therapy with cFLIP inhibitors may be an effective strategy for overcoming R27T resistance.

## INTRODUCTION

Interferon-β (IFN-β) has emerged as a potential anti-cancer drug that can effectively induce cancer growth arrest by decreasing cell proliferation and inducing apoptosis [1]. However, the use of IFN-β in anti-cancer therapy has been hampered by its low stability and relatively short circulating half-life (3-5 h in humans) [2].

In a previous study, we developed R27T, a glycoengineered version of recombinant human IFN-β1a. R27T has two N-glycosylation sites: at the 80^th^ amino acid (the original site) and an additional site at the 25^th^ amino acid, induced by mutating an arginine to threonine at the 27^th^ position [3]. R27T exhibited superior stability, solubility, productivity, and pharmacokinetic properties without any loss of biological activity or alteration of receptor binding affinity, compared to those of its parent molecule, IFN-β1a. However, the anti-cancer efficacy of R27T has not yet been fully explored. In addition, IFN-β resistance reportedly develops in diverse tumor cells via deregulation of the IFN-β signaling pathway [4–6]. Therefore, we need to explore additional approaches, such as combination therapy, as a means to enhance the therapeutic efficacy of R27T.

In this study, the anti-cancer efficacy of R27T was first examined in various cancer cells. We found that the resistance to R27T-induced anti-proliferation and apoptosis was due to the increased expression of cellular FLICE-like inhibitory protein (cFLIP), which blocks the initiation of caspase activation. We investigated whether the inhibition of cFLIP could facilitate R27T-induced caspase activation. Therefore, we evaluated the ability of cFLIP small interfering RNA (siRNA) or 4,5,6,7-tetrabromobenzotriazole (TBB, a casein kinase-2 [CK-2] inhibitor) to enhance the anti-cancer effects of R27T *in vitro* and *in vivo*.

## RESULTS

### R27T induces anti-proliferation and apoptosis

**Figure 1:**
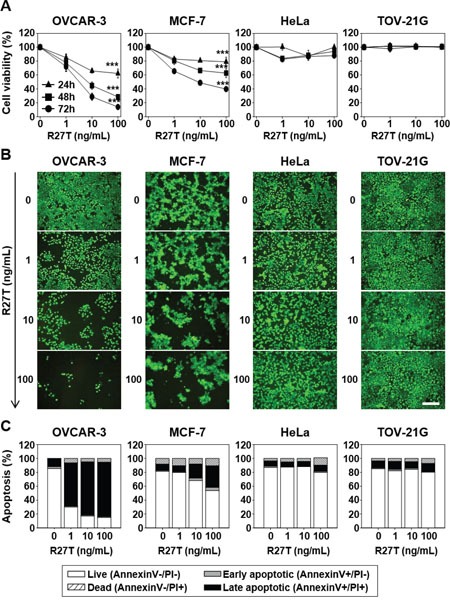
Anti-proliferative and pro-apoptotic effects of R27T in different cell lines OVCAR-3, MCF-7, HeLa, or TOV-21G cells were treated with or without various concentrations of R27T. **A**. The cells were incubated for various times, and cell viability was measured using the WTS assay. Data are presented as the means ± SD of three independent experiments (***P < 0.001 compared to the untreated group). **B**. Seventy-two hours after treatment, live cells were incubated with calcein-AM and analyzed by microscopy for detection of fluorescence. Scale bar = 250 μm. **C**. Apoptosis activity was quantified by bivariate flow cytometry of cells stained with Annexin V/PI at 72 h post-treatment.

To investigate the anti-cancer efficacy of R27T *in vitro*, we carried out two different assays in OVCAR-3, MCF-7, HeLa, and TOV-21G cells. The cell proliferation assay revealed that R27T showed a concentration-dependent anti-proliferative effect in OVCAR-3 and MCF-7 cells, but not in HeLa or TOV-21G cells (Figure 1A). Treatment of OVCAR-3 and MCF-7 cells with R27T decreased cell viability by 86.1 ± 2.1 and 60.4 ± 1.7% at 72 h post-treatment, respectively. In contrast, R27T did not significantly induce anti-cancer activity in HeLa cells compared with that in control cells. The live fluorescence assay system yielded similar results, which showed that R27T exerted notable cell-killing effects in OVCAR-3 and MCF-7 cells, but not in HeLa or TOV-21G cells (Figure 1B).

Next, we examined whether R27T could induce apoptosis in OVCAR-3, MCF-7, HeLa, and/or TOV-21G cells. We found that the treatment of OVCAR-3 and MCF-7 cells with R27T increased the proportion of late-stage apoptotic cells to 78.3 and 40.0%, respectively, compared to less than 15% in the untreated cells. However, R27T treatment did not alter the population of late-stage apoptotic cells in HeLa or TOV-21G cells (Figure 1C). Thus, our results suggest that R27T exhibits differential anti-proliferative and pro-apoptotic effects in R27T-sensitive cells (OVCAR-3 and MCF-7) versus R27T-resistant cells (HeLa and TOV-21G).

### Induction of apoptotic mediators does not correlate with R27T resistance

**Figure 2:**
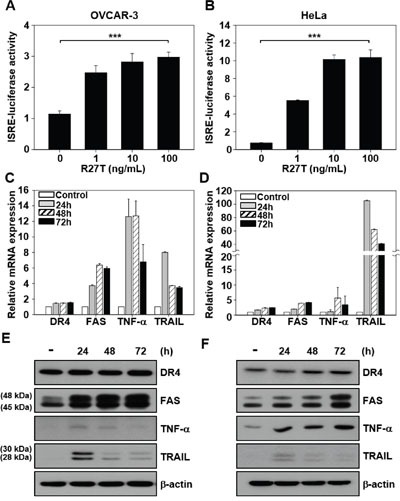
Ability of R27T to increase the expression of apoptotic mediators **A, B**. ISRE-luciferase-expressing OVCAR-3 (A) and HeLa cells (B) were treated with various concentrations of R27T for 16 h, and the ISRE luciferase activity was determined using a dual-luciferase kit. **C-F**. OVCAR-3 (C, E) or HeLa cells (D, F) were treated with R27T (100 ng/mL) for the indicated times, and the mRNA and protein levels were analyzed by qRT-PCR and western blot analysis, respectively. β-actin was used as the loading control. Error bars indicate the SD of experiments performed in triplicate (***P < 0.001 compared to the untreated group).

To investigate whether R27T resistance is related to IFN-induced signaling, we transiently transfected OVCAR-3 and HeLa cells with the interferon-sensitive response element (ISRE) luciferase reporter plasmid. R27T treatment (100 ng/mL) resulted in 2.7- and 14.9-fold increase of ISRE luciferase activity in R27T-sensitive OVCAR-3 cells (Figure 2A) and R27T-resistant HeLa cells (Figure 2B), compared with their respective controls.

To examine whether R27T resistance was correlated with the induction of apoptotic mediators, we compared the mRNA and protein expression levels of death receptor 4 (DR4), FAS, tumor necrosis factor-α (TNF-α), and TNF-related apoptosis-inducing ligand (TRAIL) in R27T-sensitive OVCAR-3 and R27T-resistant HeLa cells. qRT-PCR and western blotting analysis showed that the mRNA (Figure 2C and 2D) and protein (Figure 2E and 2F) expression levels of FAS, TNF-α, and TRAIL increased in both cell lines following R27T treatment. As shown by the results of western blot analysis, two sizes of FAS or TRAIL proteins were detected at 45/48 or 28/30 kDa, respectively (Figure 2E and 2F). Previous reports showed that two molecular weights of FAS protein resulted from different glycosylation patterns [7], and TRAIL existed in two isoforms [8]. Overall, these results indicate that R27T resistance is not associated with changes in IFN-induced signal transduction or the induction of apoptotic mediators.

### R27T-resistant cancer cells exhibit increased cFLIPS expression and blockade of caspase activation

**Figure 3:**
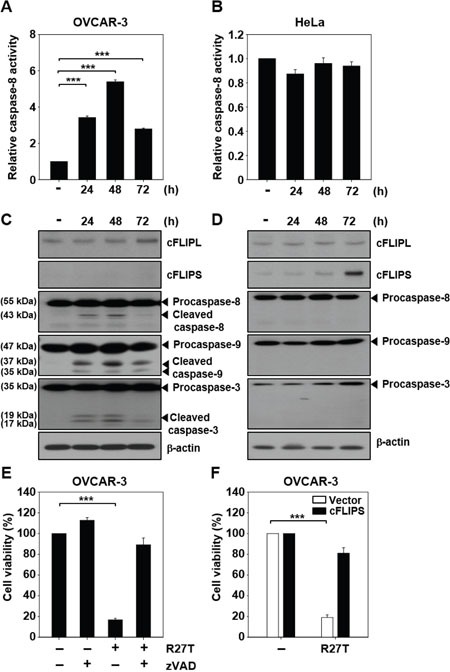
Ability of R27T to activate the caspase cascade The cells were treated with 100 ng/mL R27T and collected at the indicated times post-treatment. **A, B**. Caspase-8 activity was assessed by protease activity assays in OVCAR-3 (A) or HeLa (B) cells (***P < 0.001 compared to the untreated group). **C, D**. For the detection of caspase-3/8/9, cFLIPL, and cFLIPS, western blot analysis was performed in OVCAR-3 (C) or HeLa cells (D). β-actin was used as the loading control. **E**. OVCAR-3 cells were treated with R27T (100 ng/mL) alone or in combination with 50 μM zVAD for 72 h, and cell viability was measured by WTS assays. Data are presented as the means ± SD of three independent experiments (***P < 0.001 compared to the untreated group). **F**. OVCAR-3 cells transfected with control or cFLIPS vector were treated with 100 ng/mL R27T for 72 h. Cell viability was analyzed by WTS assays (***P < 0.001 compared to the untreated group).

To test whether changes in caspase activity contribute to R27T resistance, we examined caspase-8 activity in R27T-sensitive OVCAR-3 cells and R27T-resistant HeLa cells. Following R27T treatment, caspase-8 activity significantly and time-dependently increased up to 48 h post-treatment in OVCAR-3 cells (to 5.4-fold the basal activity; Figure 3A), but not in HeLa cells (Figure 3B). Western blotting analysis of caspase-8, -9, and -3 showed that these proteins were cleaved following R27T treatment of R27T-sensitive OVCAR-3 cells (Figure 3C) but not R27T-resistant HeLa cells (Figure 3D). These results demonstrated that the activation of caspase cascade might be blocked in R27T-resistant cells.

cFLIP is a crucial negative regulator of caspase-8 activation [9]. Therefore, we next examined the expression level of cFLIP in R27T-sensitive and -resistant cells. Increased expression of cFLIPL and cFLIPS was observed in HeLa cells compared to that in OVCAR-3 cells without R27T treatment. cFLIPS was not detected in OVCAR-3 cells (Supplementary Figure 1). Notably, there was no difference in the expression level of cFLIP protein in OVCAR-3 cells after R27T treatment compared with that in the control cells (Figure 3C). In contrast, cFLIPS protein expression was significantly increased in R27T-treated HeLa cells at 72 h (Figure 3D). To substantiate these results, we sought to test whether the late increase of cFLIPS level is related to transcription factors such as NF-κB and NFAT. We found that R27T treatment of both OVCAR-3 and HeLa cells did not significantly alter the NF-κB p65 levels for 72 h (Supplementary Figure 2). However, the activation of p65 (phosphorylated p65) and NFAT1c (dephosphorylated NFAT1c) was greater in HeLa cells (Supplementary Figure 2B) than in OVCAR-3 cells (Supplementary Figure 2A). These results revealed that the late expression of cFLIPS in R27T-resistant cells might be influenced by the increased activation of p65 and NFAT1c following R27T treatment.

The co-treatment of OVCAR-3 cells with the pan-caspase inhibitor, zVAD, recovered cell viability by 89.1 ± 6.6% compared to less than 20% observed in OVCAR-3 cells treated with R27T alone (Figure 3E). We ascertained whether the overexpression of cFLIPS plays a role in apoptosis resistance. When OVCAR-3 cells transfected with cFLIPS expression vector was treated with R27T, cell viability was restored to 81 ± 5.2% (Figure 3F). Taken together, these results show that an increase in cFLIPS expression contributes to the impairment of caspase activation in R27T-resistant cells.

### Inhibition of cFLIP expression facilitates caspase activation

**Figure 4:**
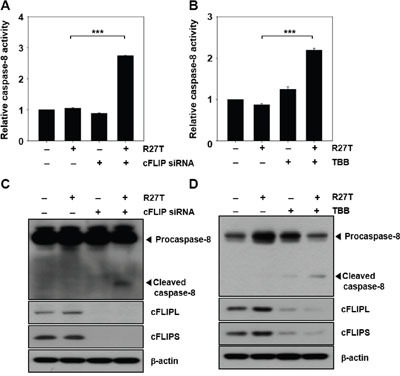
Inhibition of cFLIP expression enhances caspase activity in R27T-resistant cells **A-D**. HeLa cells were transfected with 10 nM scrambled or cFLIP siRNA for 24 h, and then treated with R27T (100 ng/mL) for additional 48 h. Cell lysates were analyzed by protease activity assays (A) or western blot analysis (C). HeLa cells were treated with 10 μM TBB in the absence or presence of R27T (100 ng/mL) for 48 h, and cell lysates were analyzed by protease activity assays (B) or western blot analysis (D). Data are presented as the means ± SD of three independent experiments (***P < 0.001 compared to the untreated group). β-actin was used as the loading control.

We further verified the effect of suppression of cFLIP on caspase-8 activity. Caspase-8 activity in HeLa cells treated with cFLIP siRNA plus R27T was 2.7-fold higher than that in HeLa cells treated with R27T alone (Figure 4A), and we detected the cleaved caspase-8 product (Figure 4C). A previous study reported that inhibition of CK-2 by TBB decreased the endogenous cFLIP levels via proteasome-mediated degradation of cFLIP, leading to the enhancement of TRAIL and FAS sensitivity in endometrial carcinoma cells [10]. Therefore, HeLa cells were co-treated with R27T and TBB, which resulted in the reduction of cFLIP expression. Furthermore, co-treatment of R27T with TBB resulted in 2.3-fold increase in caspase-8 activity (Figure 4B) and the cleavage of caspase-8 (Figure 4D). Immunoprecipitation of cFLIPL and cFLIPS with an anti-caspase-8 antibody revealed that TBB treatment did not inhibit cFLIP binding with caspase-8 but only decreased cFLIP expression, thereby activating caspase-8 on R27T co-treatment (Supplementary Figure 3).

### Inhibition of cFLIP expression sensitizes R27T-resistant cancer cells to anti-proliferative and pro-apoptotic effects

**Figure 5:**
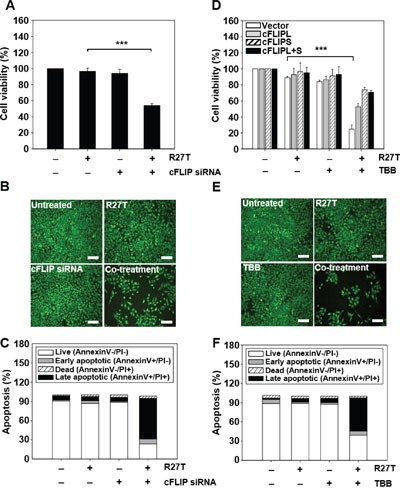
Inhibition of cFLIP expression enhances R27T-induced anti-proliferation and apoptosis in R27T-resistant cells **A-C**. HeLa cells were transfected with 10 nM of scrambled or cFLIP siRNA for 24 h, and then treated with R27T (100 ng/mL) for an additional 48 h. Cell viability was analyzed by WTS assays (A) or live-cell staining (B), and apoptosis was assessed by Annexin V/PI staining (C). **D**. HeLa cells transfected with control, cFLIPL, cFLIPS, or cFLIPL + S vector were treated with TBB (10 μM) or R27T (100 ng/mL) or both for 48 h. Cell viability was analyzed by WTS assays. **E-F**. HeLa cells treated solely with TBB (10 μM) or R27T (100 ng/mL) or both for 48 h were stained with calcein-AM (E), and apoptosis was assessed by Annexin V/PI staining (F). Error bars indicate the SD of experiments performed in triplicate (***P < 0.001 compared to the R27T-treated group). Scale bar = 250 μm.

We next examined whether the cFLIP siRNA- or TBB-mediated inhibition of cFLIP expression could enhance the anti-cancer efficacy of R27T *in vitro*. In the WST assay system, combined treatment of R27T and cFLIP siRNA significantly reduced HeLa cell viability to 54.1 ± 2.5% (Figure 5A). For the establishment of cFLIP overexpression in HeLa cells, cFLIPL, cFLIPS, or cFLIPL + S vectors were transfected. Western blot assay showed an increase in cFLIP protein expression (Supplementary Figure 4). The anti-cancer activity of R27T plus TBB was 2.1-, 3.0-, or 2.9-fold greater in control vector-transfected HeLa cells than in HeLa cells transfected with cFLIPL, cFLIPS, and cFLIPL + S vector, respectively (Figure 5D). In control vector-transfected HeLa cells, R27T plus TBB exerted 3.6-fold greater cell-killing effect compared with that of free R27T. Similarly, fluorescent microscopy revealed that the smallest live-cell populations were observed in cultures co-treated with R27T and cFLIP siRNA (Figure 5B) or TBB (Figure 5E). Moreover, apoptosis was significantly increased when HeLa cells were co-treated with R27T and cFLIP siRNA or TBB, with the late apoptotic cell populations increasing to 63.5 (Figure 5C) or 51.6% (Figure 5F) compared to less than 10% observed in the controls, respectively. These results suggest that the combined treatment of R27T and a cFLIP inhibitor can overcome R27T resistance and increase the therapeutic efficacy of R27T in resistant cells.

### Combined treatment with R27T and TBB suppresses *in vivo* tumor growth

**Figure 6:**
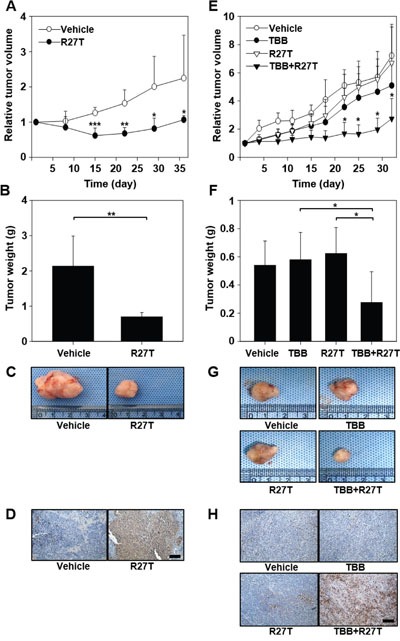
*In vivo* anti-tumor efficacy of co-treatment with R27T and TBB **A-D**. OVCAR-3-tumor-bearing mice were intraperitoneally treated with vehicle or R27T (1 mg/kg) three times per week for 3 weeks (n = 5 per group). Tumor volumes were measured in each group (A). OVCAR-3 tumor tissues were extracted for weight analysis (B) and visualization (C), and sectioned for TUNEL staining (D). **E-H**. HeLa tumor-bearing mice were intraperitoneally treated three times per week for 4 weeks with either vehicle or TBB (10 mg/kg), and then with R27T (1 mg/kg; n = 5 per group). Tumor volumes were periodically measured using calipers (E). HeLa tumor tissues were extracted for weight analysis (F) and visualization (G), and were sectioned for TUNEL staining (H). *P < 0.05 in comparison with the other groups, as analyzed by the Student-Newman-Keuls test and ANOVA. Scale bar = 100 μm.

We next examined the *in vivo* anti-tumor efficacy of R27T in an OVCAR-3 tumor xenograft model. The tumor volumes were significantly smaller (Figure 6A) and tumor weights were lower (Figure 6B and 6C) in mice treated with R27T than in those treated with the control vehicle. The tumor weight of R27T-treated group was 3.1-fold lower than that of the vehicle. When we further examined the anti-tumor effects of intraperitoneally co-administered R27T and TBB in HeLa tumor-bearing mice, significant inhibition of tumor growth was observed in the R27T/TBB co-treated group (Figure 6E). The tumor weights were lowest in the mice co-treated with R27T and TBB, which exhibited a 3-fold reduction in tumor weight compared with that of R27T-treated mice (Figure 6F and 6G). In keeping with these tumor-growth inhibition effects, terminal deoxynucleotidyl transferase dUTP nick end labeling (TUNEL) analysis showed population of apoptotic cells in OVCAR-3 tumor tissues of R27T-treated mice (Figure 6D) or HeLa tumor tissues of R27T plus TBB- treated mice (Figure 6H) was higher than that in the control-treated group. Furthermore, a significant decrease in cFLIP staining was observed in HeLa tumor tissues of R27T plus TBB-treated mice (Supplementary Figure 5). Collectively, these results indicate that the anti-tumor activity of R27T could be enhanced by combination treatment with TBB.

## DISCUSSION

In this study, we demonstrate the concentration-dependent anti-proliferative and pro-apoptotic effects of R27T in OVCAR-3 and MCF-7 cells via significant induction of IFN and death receptor signaling pathways. In contrast, R27T did not exhibit significant anti-cancer activity in HeLa cells, in which the overexpression of cFLIPS was associated with impaired caspase-8 activity and R27T resistance. Moreover, co-treatment with R27T and a cFLIP inhibitor was found to reduce the growth of R27T-resistant cells *in vitro* and *in vivo*.

It is notable that R27T has significant anti-cancer effects in OVCAR-3 and MCF-7 cells (Figure 1A) because several previous reports have described the anti-proliferative effects of IFN-β in OVCAR-3 and MCF-7 cells [11, 12]. Consistent with our results, previous reports showed that IFN-β treatment did not induce apoptosis in HeLa cells [12, 13]. TOV-21G cells have a Janus kinase (JAK1)-truncating mutation [14], which suggests that R27T does not induce IFN signaling, enabling it to exert a dominant-negative effect on apoptosis (Figure 1C).

Unlike the nature of its anti-cancer effects, R27T increased ISRE luciferase activity and expression levels of apoptotic mediators in both OVCAR-3 and HeLa cells (Figure 2). It has been reported that IFN-stimulated gene factor-3 (ISGF-3) can bind to ISRE in OVCAR-3 or HeLa cells treated with type I IFNs [15, 16]. Furthermore, the IFN-β-induced expression of interferon-stimulated genes (ISGs; e.g., DR4, FAS, TNF-α, and TRAIL) has been correlated with death receptor-mediated activation of apoptotic signals [17–20]. Regardless of the activation of IFN and death receptor signaling pathways, there were significant differences in caspase activity between R27T-treated sensitive and -resistant cell lines (Figure 3A-3D). The caspase cascade can be initiated through either intrinsic (mitochondrial-mediated) or extrinsic (death receptor-mediated) apoptosis pathway. The extrinsic apoptosis pathway is triggered by ligand binding to death receptors, resulting in caspase-8 activation. Activated caspase-8 then directly cleaves effector caspases (caspase-3, -6, -7) or activates the intrinsic apoptotic pathway via cleavage of Bcl-2 homology (BH3)-interacting domain death agonist, resulting in caspase-9 activation. Activated caspase-9 then triggers apoptosis by activating effector caspases [21]. In this study, we found that the anti-cancer efficacy of R27T in OVCAR-3 cells was inhibited by the pan-caspase inhibitor, zVAD, which prevented R27T-mediated anti-proliferative effects (Figure 3E), and has been used to study the mechanisms underlying the effects of IFN-β in various cancer cell lines [22, 23].

In the absence of R27T, cFLIPL and cFLIPS proteins were more highly expressed in R27T-resistant cells than in R27T-sensitive cells, thereby blocking caspase-8 activation (Supplementary Figure 1 and Figure 3D). Moreover, when HeLa cells were treated with R27T for 72 h, an increase in cFLIPS expression was observed (Figure 3D). cFLIP is known to be a major anti-apoptotic regulator and resistance factor that inhibits death receptor-induced apoptosis in a wide range of human malignancies [24, 25]. Recruitment of cFLIP to death-inducing signaling complex (DISC) prevents the processing and activation of procaspase-8 [24, 25]. Although 15 different isoforms of cFLIP have been currently identified, cFLIPL and cFLIPS are the major anti-apoptotic proteins [26, 27]. cFLIPL is structurally similar to procaspase-8 but lacks the catalytic cysteine residue and proteolytic activity, while cFLIPS only contains two N-terminal death effector domains [28]. In addition, cFLIPL forms heterodimeric complex with procaspase-8, resulting in the partial activation of caspase-8. Recruitment of cFLIPS to the DISC impedes homodimerization and activation of procaspase-8, preventing the downstream apoptosis cascade [29]. Notably, in an earlier report, TRAIL-induced upregulation of cFLIPS was shown to increase the survival of non-small cell lung carcinoma cells by reducing TRAIL sensitivity, whereas the cFLIPL isoform did not appear to be involved in TRAIL-induced apoptosis [30]. A previous report showed that cFLIPL triggered FAS-induced apoptosis upon strong receptor stimulation, while the overexpression of cFLIPS inhibited caspase-8 activation even upon expanded stimulation of FAS receptor [31]. Previous reports provided evidence that cFLIPS expression was significantly upregulated 72 h after TRAIL treatment [30], and cFLIPS expression was linked to the calcineurin/NFAT pathway in T-cells [32]. NFAT can bind to cFLIP promoter, thereby promoting the expression of cFLIPS [32]. Activation of NF-κB enhanced histone deacetylase 1, reduced p300 and histone acetylation, and prevented the recruitment of NFAT to cFLIP promoter, resulting in the transcriptional repression of cFLIP [33]. The results of this study as well as previous studies suggest that R27T resistance of tumor cells might result from increased cFLIPS expression.

Downregulation of cFLIP by siRNA or TBB rendered R27T-resistant cells sensitive to R27T-mediated apoptosis and was accompanied by cleavage of caspase-8 (Figure 4 and 5). Since the upregulation of cFLIP impairs caspase-8 activation, siRNA-mediated silencing of cFLIP could logically be predicted to promote the apoptosis of R27T-resistant cells. A previous report showed that the downregulation of cFLIP gene expression increased the pro-apoptotic effect of type I IFNs [34]. In addition, co-treatment with TRAIL and a cFLIP-targeting siRNA has been recently reported to enhance the apoptosis of TRAIL-resistant tumor cells via caspase-8 activation [35, 36]. Moreover, combined treatment with TRAIL and TBB was shown to increase the TRAIL sensitivity on cancer cells [37, 38]. Thus, we chose cFLIP siRNA and CK-2 inhibitor, TBB, for combination therapy with R27T.

In conclusion, this study is the first to show that the induction of apoptosis in cancer cells by R27T involves a cFLIP-mediated mechanism. We further suggest that it may be possible to enhance the therapeutic efficacy of R27T significantly by downregulating cFLIP. Thus, combination therapy with R27T and cFLIP inhibitors may be an attractive strategy for overcoming R27T resistance.

## MATERIALS AND METHODS

### Construction of plasmids

In order to establish a cell line that transiently express cFLIP-short (cFLIPS), pCMV3-cFLIPS was constructed. The cFLIPS gene synthesized by Bioneer (Daejeon, Korea) was excised with KpnI (Thermo Scientific, Pittsburgh, PA, USA) and XbaI (Thermo Scientific) and inserted to pCMV3. The pCMV-cFLIP-long (cFLIPL) was purchased by Sino Biologinal (Beijing, China).

### Cell lines and culture condition

Human ovarian carcinoma (OVCAR-3, TOV-21G), human cervical cancer (HeLa), and human breast cancer (MCF-7) cell lines were purchased from the American Type Culture Collection (ATCC; Manassas, VA, USA). OVCAR-3, HeLa, and MCF-7 cells were cultured in RPMI-1640 (HyClone, Logan, UT, USA) supplemented with 10% fetal bovine serum (HyClone), 100 units/mL penicillin, and 100 μg/mL streptomycin (HyClone). TOV-21G cells were maintained in a 1:1 mixture of M199/MCDB medium (HyClone) containing 15% fetal bovine serum, 100 units/mL penicillin, and 100 μg/mL streptomycin. All cells were grown at 37°C in a humidified 5% CO_2_ atmosphere.

### Cell viability and apoptosis assays

The *in vitro* anti-proliferation efficacy of R27T (provided from Abion Inc. [3]) was tested using two different cell viability assays. OVCAR-3, MCF-7, HeLa, or TOV-21G cells were seeded to 96-well plates, cultured overnight, and then treated with or without various concentration of R27T for 24, 48, or 72 h. For combination treatment, OVCAR-3 or HeLa cells were treated with R27T (100 ng/mL), zVAD (50 μM; R&D Systems, Minneapolis, MN, USA), and/or TBB (10 μM; Tocris Bioscience, Bristol, UK), alone or in combination, for 48 or 72 h. ON-TARGET plus human cFLIP siRNA set was supplied by Dharmacon (Lafayette, CO, US). The sequences of siRNAs used for transfection were as follows: 5′-GUGCCGGGAUGUUGCUAUA-3′ (sense) and 5′- UAUAGCAACAUCCCGGCAC-3′ (antisense); 5′-CAAGCAGUCUGUUCAAGGA-3′ (sense) and 5′-UCCUUGAACAGACUGCUUG-3′ (antisense); 5′-CAUGGUAUAUCCCAGAUUC-3′ (sense) and 5′-GAAUCUGGGAUAUACCAUG-3′ (antisense); 5′-CCUAGGAAUCUGCCUGAUA-3′ (sense) and 5′-UAUCAGGCAGAUUCCUAGG-3′ (antisense). For sequential treatment with R27T and cFLIP siRNA, HeLa cells were transfected with 10 nM of scrambled siRNA (Dharmacon) or cFLIP siRNA for 24 h. Thereafter, the siRNA-containing culture medium was removed and cells were cultured in fresh medium containing R27T (100 ng/mL). Cell viability was assessed using a water-soluble tetrazolium (WST) colorimetric assay (Ez-Cytox; Daeil Lab Service, Seoul, Korea) or a live cell-staining assay (LIVE/DEAD Viability Assay; Molecular Probes, Eugene, OR, USA). For the WST assay, 10 μL of WST reagent was added to each well, the plates were incubated for 1 h, and absorbance was measured at 430 nm using a microplate reader (TECAN, Durham, NC, USA). For live-cell analysis, calcein-AM was treated to cells for 10 min and the fluorescence of calcein-AM (which is specifically taken up by live cells) was analyzed using a fluorescence microscope (Leica, Wetzlar, Germany).

R27T-induced apoptosis was measured using a fluorescence isothiocyanate (FITC) Annexin V/PI kit (BD Bioscience, San Jose, CA, USA). Annexin V can detect apoptotic cells by binding to phosphatidylserine and PI is used for nuclear DNA staining. Cells are Annexin V positive and PI negative in early apoptosis, while cells are both Annexin V and PI positive in late apoptosis [39]. Briefly, cells were harvested, washed twice with phosphate-buffered saline (PBS), and resuspended in 100 μL of binding buffer (10 mM HEPES/NaOH [pH 7.4], 140 mM NaCl, and 2.5 mM CaCl_2_). Five microliters of FITC Annexin V or PI solution were added to each sample, and the samples were incubated for 15 min at room temperature in the dark. The reaction was stopped by adding 400 μL of binding buffer. The stained samples were run on a FACSCalibur flow cytometer (BD Biosciences) and analyzed with the Cell QuestPro (BD Biosciences).

### Luciferase reporter assay

Reporter gene assays were performed using a Dual Luciferase Assay kit (Promega, Madison, WI, USA). OVACAR-3 or HeLa cells were seeded to 12-well plates and incubated overnight at 37°C in a humidified atmosphere containing 5% CO_2_. The cells were then co-transfected with 0.4 μg of a firefly luciferase-encoding reporter plasmid containing the interferon stimulated response element (ISRE) and 0.3 μg of pTK-RLuc plasmid containing the *Renilla* luciferase reporter gene. Transfections were performed with the Lipofectamine LTX reagent (Invitrogen, Carlsbad, CA, USA). At 24 h post-transfection, cells were treated with the indicated concentrations of R27T, and after an additional 16 h, the activities of firefly luciferase and *Renilla* luciferase were determined using a microplate reader (TECAN).

### Quantitative real-time reverse transcription-polymerase chain reaction (qRT-PCR)

OVCAR-3 or HeLa cells were treated with 100 ng/mL R27T, total RNA was isolated using a Hybrid-RTM kit (GeneAll, Seoul, Korea), and the RNA was reverse transcribed to complementary DNA (cDNA) using a Transcriptor First Strand cDNA synthesis kit (Roche, Indianapolis, IN, USA). TaqMan qRT-PCR was performed using the following primers and probes: DR4 (forward primer, 5′-GGGTCCACAAGACCTTCAAGT-3′; reverse primer, 5′-TGCAGCTGAGCTAGGTACGA-3′; and probe, 5′-FAM-TCCTGCTG-TAMRA-3′), FAS (forward primer, 5′-ATGGCCAATTCTGCCATAAG-3′; reverse primer, 5′-TGACTGTGCAGTCCCTAGCTT-3′; and probe, 5′-FAM-TCCTCCAG-TAMRA-3′), TNF-α (forward primer, 5′-GACAAGCCTGTAGCCCATGT-3′; reverse primer, 5′-TCTCAGCTCCACGCCATT-3′; and probe, 5′-FAM-CCTCCTGG-TAMRA-3′), and TRAIL (forward primer, 5′-CCTCAGAGAGTAGCAGCTCACA-3′; reverse primer, 5′-CAGAGCCTTTTCATTCTTGGA-3′; and probe, 5′-FAM-CAGAGGAA-TAMRA-3′). The following cycling conditions were applied: 95°C for 10 min; followed by 50 cycles of 95°C for 10 s and 55°C for 30 s; and a final cycle at 40°C for 30 s. The primers and probes were designed by the Universal Probe Library Assay Design Center (Roche Diagnostics, Basel, Switzerland), and qRT-PCR was performed on a LightCycler 2.0 (Roche Diagnostics, Mannheim, Germany). The 2(-ΔΔC(T)) method was used to compute relative mRNA expression levels [40].

### Antibodies and western blot analysis

OVCAR-3 or HeLa cells treated with R27T (100 ng/mL), cFLIP siRNA (10 nM), and/or TBB (10 μM), alone or in combination, were lysed in RIPA lysis buffer (150 mM sodium chloride, 1% Triton X-100, 1% sodium deoxycholate, 0.1% SDS, 50 mM Tris-HCl [pH 7.5], 2 mM EDTA) supplemented with a protease inhibitor mixture (Roche). The lysed cells were incubated for 20 min on ice and clarified by centrifugation at 12,000 × g for 15 min. The supernatant was collected, and protein concentration was determined using a BCA protein assay kit (Thermo Scientific) according to the manufacturer's instructions. Twenty micrograms of protein were resolved by 12% SDS-PAGE and transferred to a polyvinyl difluoride membrane (PVDF; BioRad, CA, USA). The PVDF membranes were blocked in TBS-Tween20 buffer containing 5% skim milk and incubated with primary antibodies at 4°C overnight. The following primary antibodies were utilized: anti-FAS, anti-TNF-α, anti-TRAIL, anti-caspase-8, anti-caspase-3, anti-caspase-9, anti-p65, anti-p-p65 (all from Cell Signaling Technology, Danvers, MA, USA), anti-cFLIPS (AG Scientific, CA, USA), anti-DR4 (Abcam, Cambridge, UK), anti-cFLIPL, anti-nuclear factor of activated T-cells 1c (NFAT1c) and anti-β-actin (both from Santa Cruz Biotechnology, Santa Cruz, CA, USA). The blots were then incubated for 1 h with anti-mouse IgG-HRP (ThermoFisher Scientific) or anti-rabbit IgG-HRP (ThermoFisher Scientific) secondary antibodies, and immunoreactive proteins were visualized using enhanced chemiluminescence reagents (GE Healthcare). For immunoprecipitation, cells were lysed in lysis buffer containing 20 mM Tris [pH 7.5], 150 mM NaCl, 1% NP-40, 10% glycerol, and protease inhibitor mixture (Roche) for 1h. The lysed cells were clarified by centrifugation at 12,000 × g for 15 min. The supernatant was collected and protein concentration was determined using a BCA protein assay kit (Thermo Scientific) according to the manufacturer's instructions. One milligram of protein was precleared in the presence of 20 μl Dynabead protein G (Thermo Scientific) for 1h at 4°C. After centrifugation, the supernatant was collected and incubated with 1 μg of anti-caspase-8 antibody (Acris Antibodies, Herford, Germany) in the presence of 20 μL Dynabead protein G (Thermo Scientific). The precipitated beads were washed 3 times in lysis buffer and then the precipitated proteins were analyzed by Western blotting.

### Caspase-8 activity analysis

Caspase-8 activity was measured using a Caspase-Glo 8 assay kit (Promega). OVCAR-3 or HeLa cells were treated with R27T (100 ng/mL), cFLIP siRNA (10 nM), and/or TBB (10 μM) alone or in combination, and then lysed on ice in phosphate buffered saline containing 1% NP40 and 0.1% SDS. Ten micrograms of cell lysate in a 50 μL total volume was resuspended in 50 μL of Caspase-Glo reagent and incubated for 1 h at room temperature. The luminescence produced upon cleavage of the luminogenic substrate (which contained the caspase-8 cleavage site, LETD) was measured for each sample using a microplate reader (TECAN).

### Assessment of *in vivo* anti-tumor efficacy

The anti-tumor efficacy of R27T *in vivo* was tested in OVCAR-3 and HeLa xenograft models. All animal experiments were approved by the Institutional Animal Care and Use Committee (IACUC) of Seoul National University (SNU-160310-1). Five-week-old female athymic nude mice (Orient Bio, Seongnam, Gyeonggi, South Korea) were subcutaneously inoculated at the dorsal right side with 5 × 10^6^ cells. OVCAR-3-tumor-bearing mice were intraperitoneally treated with R27T (1 mg/kg) every other day for three weeks. In HeLa xenograft model, the mice were intraperitoneally administered three times per week for 4 weeks with either vehicle or TBB (10 mg/kg) or/and R27T (1 mg/kg). In the co-treatment group, mice were treated with TBB and R27T alternately every day for 4 weeks. Tumor size was measured by caliper every other day in two dimensions, and the tumor volume was calculated using the following formula; tumor volume (mm^3^) = (short diameter)^2^ x (long diameter) x 0.5. For histological evaluation, tumor tissues were extracted, weighed, sectioned, and stained with TUNEL. In addition, expression level of cFLIP was determined by immunohistochemical staining HeLa tumor tissue section (4 μm thick) with an anti-cFLIP antibody (AG Scientific).

### Statistics

Statistics were conducted by analysis of variance (ANOVA), with the Student-Newman-Keuls test. The SigmaStat software (Systat Software, Richmond, CA, USA) was employed, and *P* < 0.05 was considered statistically significant.

## SUPPLEMENTARY FIGURES


