# Supplementary material for: Sensitization of glycoengineered interferon-β1a-resistant cancer cells by cFLIP inhibition for enhanced anti-cancer therapy
Source: Oncotarget. 2017 Jan 10;8(8):13957–70. doi: 10.18632/oncotarget.14573 (PMC5355153; doi:10.18632/oncotarget.14573)
Supplement: Supplementary file 1 [file oncotarget-08-13957-s001.pdf]

Sensitization of glycoengineered interferon-β1a-resistant cancer cells by cFLIP inhibition for enhanced anti-cancer therapy

SUPPLEMENTARY FIGURES

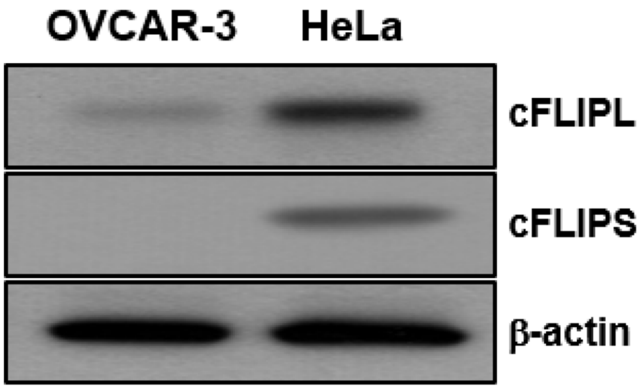

Supplementary Figure 1: cFLIP expression in R27T-sensitive and -resistant cancer cells. Protein expression of cFLIPL and cFLIPS was analyzed using western blotting in the absence of R27T. β-actin was used as the loading control.

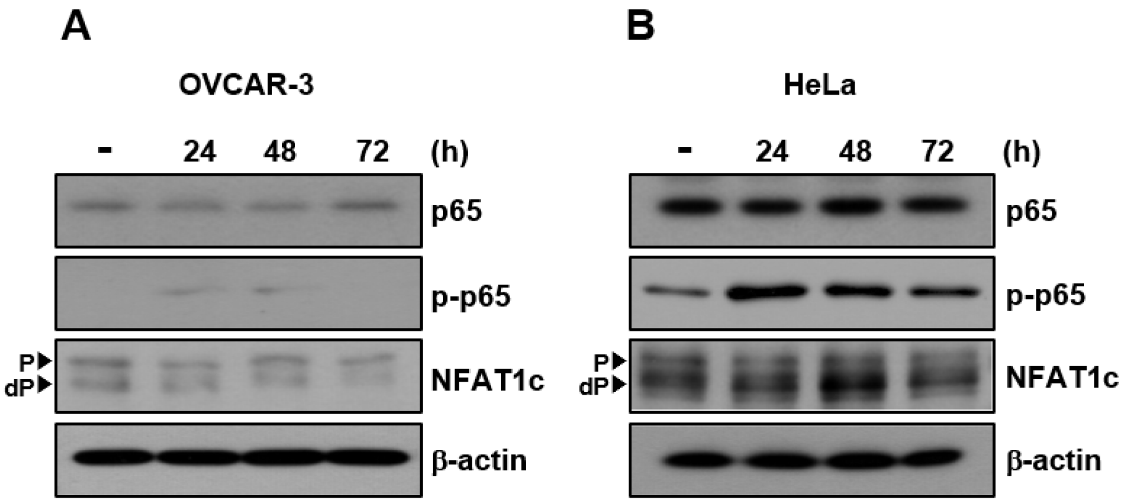

Supplementary Figure 2: Activation of NF-κB p65 and NFAT1c by R27T treatment. A, B. OVCAR-3 (A) and HeLa (B) cells were treated with 100 ng/mL of R27T for the indicated time. For detection of p65, p-p65, and NFAT1c, western blot analysis was performed. β-actin was used as the loading control.

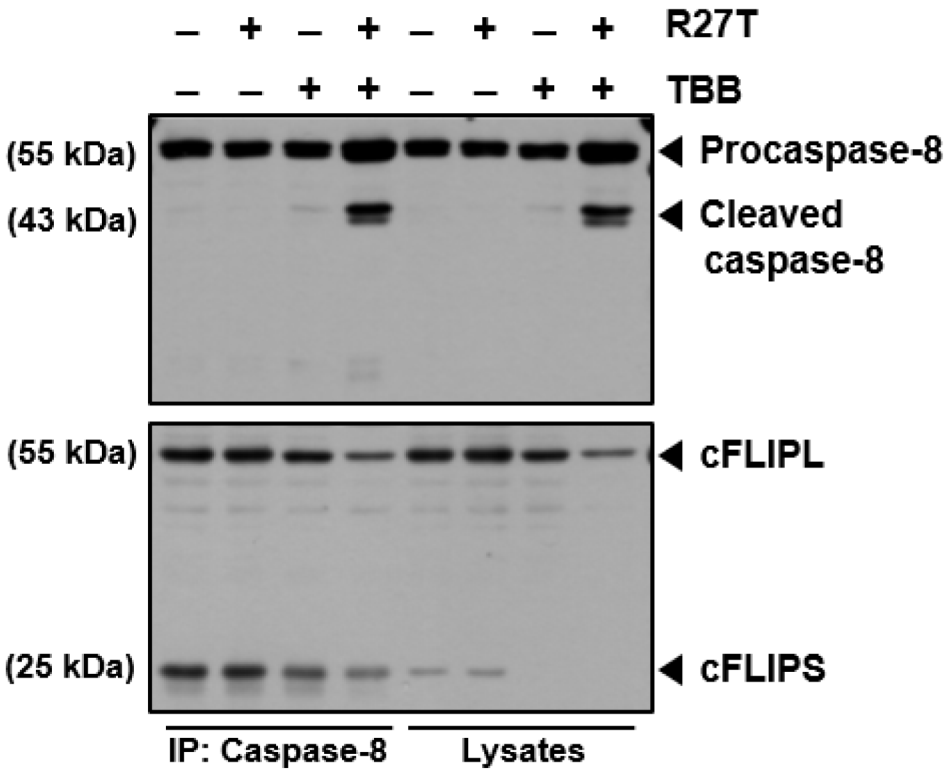

Supplementary Figure 3: TBB-mediated reduction of cFLIP. HeLa cells were treated with 10 μM TBB in the absence or presence of R27T (100 ng/mL) for 48 h, and the cell lysates were used for western blotting before (lysates) or after (IP: Caspase-8) immunoprecipitation with an anti-caspase-8 antibody.

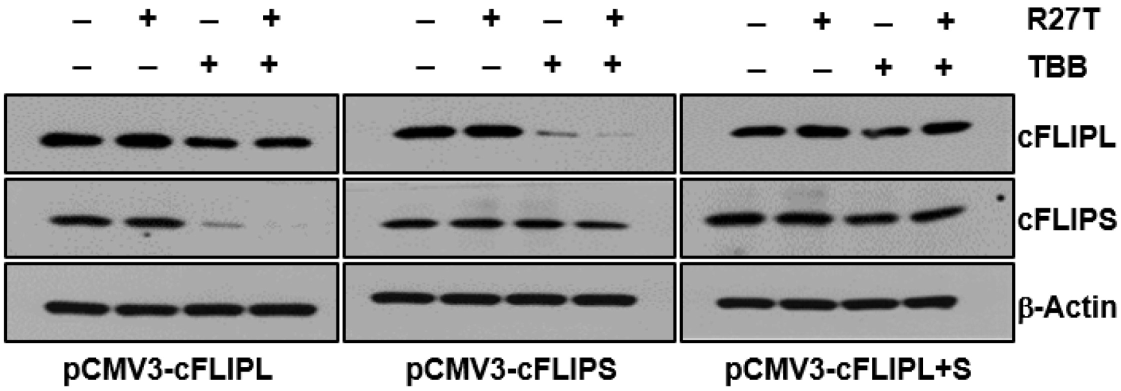

Supplementary Figure 4: Restoration of cFLIP by expression vector transfection prior to TBB or R27T treatment. HeLa cells transfected with cFLIPL, cFLIPS, or cFLIPL + S vector were treated with TBB (10 µM) or R27T (100 ng/mL) or both for 48 h. For the detection of cFLIPL and cFLIPS, western blot analysis was performed. β-actin was used as the loading control.

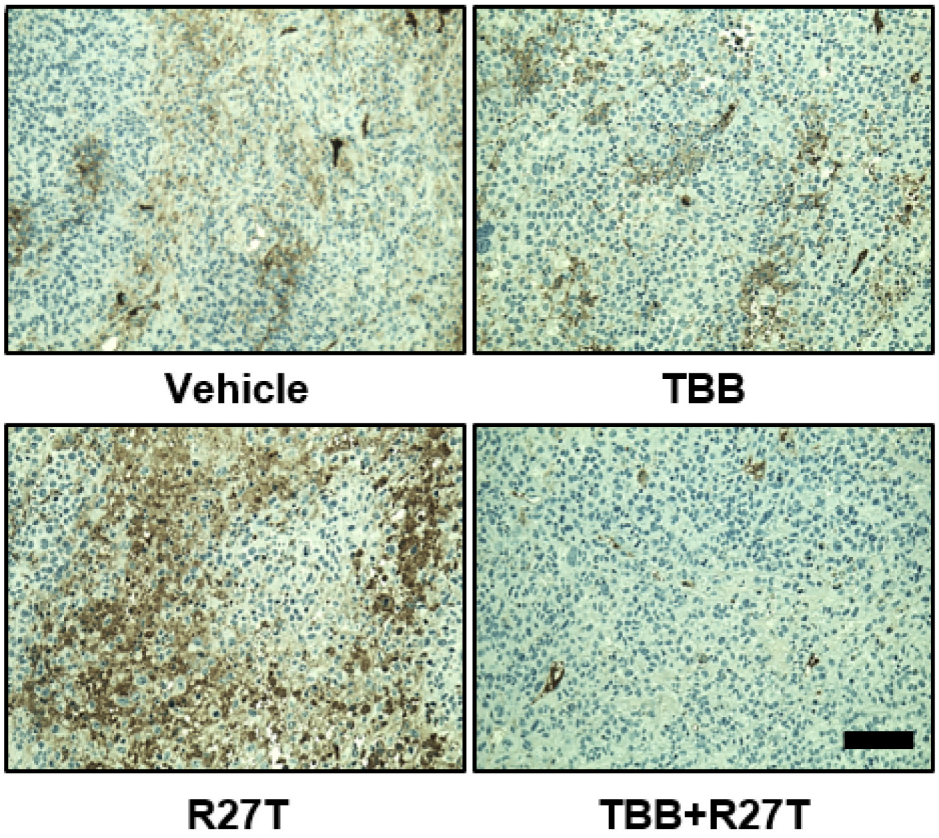

Supplementary Figure 5: Immunohistochemical detection of cFLIP in HeLa tumor xenografts. HeLa tumor-bearing mice were intraperitoneally administered with TBB (10 mg/kg), R27T (1 mg/kg), or TBB + R27T (10 mg/kg TBB, 1 mg/kg R27T) three times per week for 4 weeks. Immunohistochemical staining of the tumor tissues was performed using anti-cFLIP antibody. Scale bar = 100 µm.
